# An Unusual Presentation of Cholangiocarcinoma: The Sister Mary Joseph Nodule—A Case Report

**DOI:** 10.3390/reports9010051

**Published:** 2026-02-03

**Authors:** Toni Esposito, Niharika Singh, Riddhish Sheth, George Keckeisen

**Affiliations:** 1 NSU Kiran Patel College of Osteopathic Medicine, Davie, FL 33328, USA; 2 Department of Surgery, Renaissance School of Medicine at Stonybrook University, Stonybrook, NY 11794, USA; niharika.singh@stonybrookmedicine.edu; 3 Department of Pathology, Renaissance School of Medicine at Stonybrook Southampton, Southampton, NY 11968, USA; riddhish.sheth@stonybrookmedicine.edu; 4 Department of Surgery, Renaissance School of Medicine at Stonybrook Southampton, Southampton, NY 11968, USA; george.keckeisen@stonybrookmedicine.edu

**Keywords:** Sister Mary Joseph Nodule, cholangiocarcinoma, intra-abdominal malignancy

## Abstract

**Background and Clinical Significance**: Sister Mary Joseph nodules (SMJN) are rare extra-intestinal manifestations of metastatic intra-abdominal and pelvic malignancies, often indicating advanced disease and poor prognosis. Their association with cholangiocarcinoma (CCA) is particularly uncommon, with only a limited number of reported cases. **Case Presentation**: We present a case report of a 65-year-old previously healthy male who presented for an elective umbilical hernia repair. Additional findings of elevated direct bilirubin and a history of fatigue in the patient prompted further evaluation with biopsy and imaging, which revealed advanced-stage intrahepatic CCA. Following the diagnosis, the patient underwent biliary stenting and chemotherapy. **Conclusions**: The variable presentation of SMJN, along with its frequent misdiagnosis, often delays recogni-tion and management of the underlying malignancy. This case of SMJN in the setting of CCA highlights the complex interplay between intra-abdominal and pelvic malignancies and their impact on different organ systems. With the rising incidence and mortality rates associated with CCA, early recognition is essential to improving patient outcomes. This underscores the need for increased clinical awareness and further research, thus support-ing the development of this case report.

## 1. Introduction and Clinical Significance

Cholangiocarcinoma (CCA) is a highly aggressive malignancy that originates from epithelial cells lining the bile ducts [1]. It accounts for approximately 3% of all gastrointestinal cancers and is classified based on its anatomical location within the biliary tree [2,3]. Intrahepatic CCAs, located above second-order bile ducts, remain the second most prevalent cause of primary liver cancer [3]. Conversely, extrahepatic CCAs, found below this level, can be further divided based on their relation to the cystic duct into perihilar and distal lesions. Despite their common origin from biliary epithelial cells, these distinct subtypes exhibit great variability in clinical presentation and management [3]. Intrahepatic lesions often present as a mass and may remain asymptomatic until reaching an advanced stage. In contrast, extrahepatic lesions frequently manifest with obstructive symptoms, such as jaundice or right upper quadrant pain [4]. Due to the highly variable and nonspecific nature of its symptoms, CCA is often diagnosed at a more advanced stage. Less than one-third of cases present with solitary lesions less than 3 cm at diagnosis, approximately half exhibit regional lymph node invasion, and a quarter exhibit distant metastases [5].

Carcinogenesis of biliary epithelium is a multi-step process driven by chronic inflammation and cellular injury in combination with bile flow obstruction. This prolonged bile stasis exposes biliary cells to the carcinogenic effects of bile components [4]. Although the mechanism of carcinogenesis remains similar amongst the different types of CCA, risk factors vary considerably, with primarily infectious etiology in the east and noninfectious in the west [4]. The high incidence of exposure to liver flukes, namely Clonorchis sinensis, in the Eastern Hemisphere accounts for the majority of CCA in that region. Conversely, risk factors such as cirrhosis, hepatitis B and C, and primary sclerosing cholangitis represent potential sources of malignancy in the Western Hemisphere. Given a rising incidence of CCA in Western countries and its high mortality rate, the need to understand its pathogenesis and early diagnostic approaches becomes increasingly critical.

A Sister Mary Joseph nodule (SMJN) is a rare extra-intestinal manifestation indicative of advanced-stage intra-abdominal malignancy [2]. Named after Sister Mary Joseph, who first identified the link between periumbilical nodules and intra-abdominal cancers, the presence of this nodule should prompt immediate investigation with abdominal imaging and biopsy of the lesion [6]. Its superficial position facilitates biopsy, helping pinpoint the origin of the malignancy [2]. Although the incidence of umbilical metastasis, namely SMJN, remains low at 1–3% of all intra-abdominal or pelvic malignancies, commonly gastric, colon, or ovarian, its association with CCA is unusual, exemplifying its potential diagnostic significance [7]. Currently, there have only been ten recorded cases of CCA presenting with an SMJN, making this case report the eleventh [2].

Regarding the metastatic potential of intra-abdominal and pelvic malignancies, the mechanism of umbilical seeding from its primary tumors remains poorly understood, resulting in several hypotheses to account for the phenomenon. One theory suggests that seeding may occur through contiguous spread via peritoneal infiltration, the most common route, as well as hematogenous or lymphatic spread [8]. Another proposed mechanism involves spreading through embryonic structures. The connection of the umbilicus with embryological remnants and its ample vascular and lymphatic components facilitates the migration of tumor cells. Additionally, its lack of a muscular layer provides fewer barriers to the direct spread of peritoneal masses [9]. The presence of an SMJN across various intra-abdominal or pelvic malignancies further exemplifies the complexity of determining the mechanism behind umbilical metastasis.

The clinical presentation of an SMJN can vary greatly. Depending on the primary tumor source, it can present as a fissured or ulcerated nodule that can secrete serous, mucinous, purulent, or bloody discharge. The lesion has been observed to be variously colored, including white, bluish-violet, or brownish-red, with possible pruritus [7]. Its wide array of presentations can lead to frequent initial misdiagnosis, with differential diagnoses including omphalitis, umbilical hernia, and superficial malignant tumors [7]. In this case, the SMJN was misdiagnosed as an umbilical hernia and presented as a polypoid, cauliflower-like, skin-colored mass.

The prognosis of CCA remains poor, with a mean life expectancy ranging anywhere from 2 to 11 months without treatment. Current aggressive therapeutic approaches may extend survival rates up to 21 months [8]. Interventions include surgical resection, photodynamic therapy, systemic chemotherapy, and palliative biliary decompression stenting [4]. Despite these current advances, CCA has a dismal prognosis and often remains incurable because of its refractory nature and resistance to most currently used surgical or medical interventions [4]. Additionally, metastatic disease manifesting as an SMJN is an ominous sign of advanced disease that is not amenable to cure and often suggests that patients should focus on supportive care [2].

We present a case report of a 65-year-old male with an umbilical hernia and a cholestatic symptomatology pattern. The patient was initially set to undergo an elective open umbilical hernia repair. Abnormal presentation of the umbilical hernia suggested that the exophytic mass may be an SMJN. Imaging and pathology reports of the mass were consistent with the diagnosis of advanced-stage cholangiocarcinoma.

## 2. Case Presentation

An active 65-year-old male patient with no comorbidities presented with an umbilical lesion that was diagnosed as an umbilical hernia in the outpatient setting. He had noticed it over the past two months and requested an elective repair.

**Figure 1:**
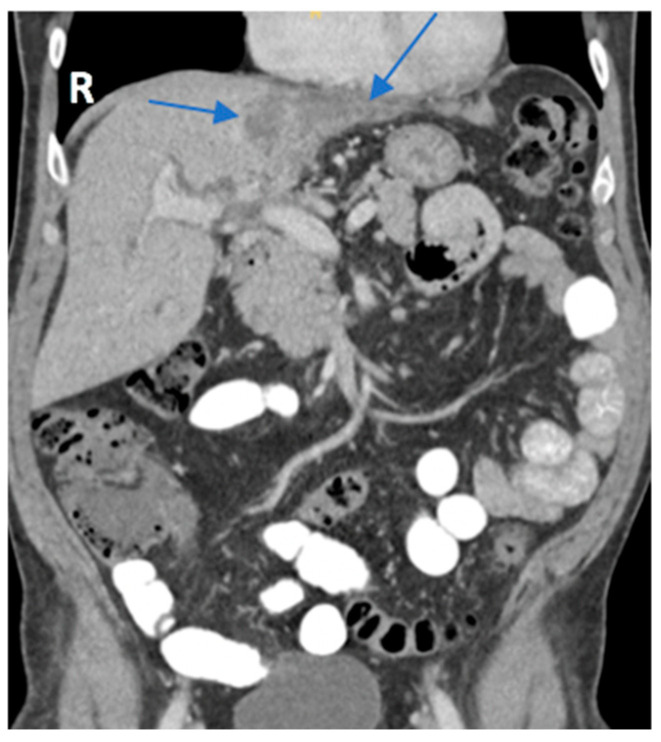
Coronal view of the CT of the abdomen and pelvis with contrast showed irregular enhancement in the right upper quadrant, suggestive of an intrahepatic cholangiocarcinoma with involvement of the left hepatic lobe (blue arrows). The primary tumor metastasized to the umbilicus and presented as an umbilical mass found on examination of the patient.

**Figure 2:**
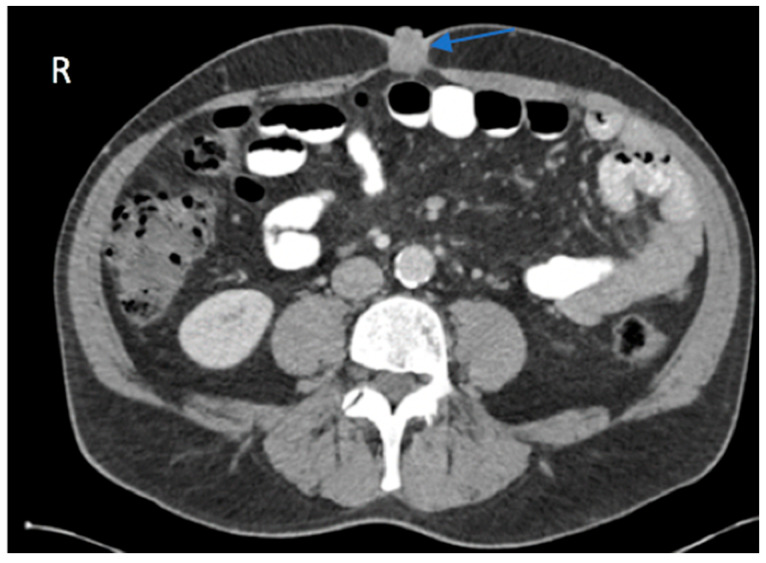
Axial view of the CT of the abdomen and pelvis with contrast showed induration at the umbilicus consistent with the location of the patient’s skin lesion (blue arrow). Biopsy of the lesion was compatible with a Sister Mary Joseph nodule, indicative of the patient’s intrahepatic cholangiocarcinoma.

**Figure 3:**
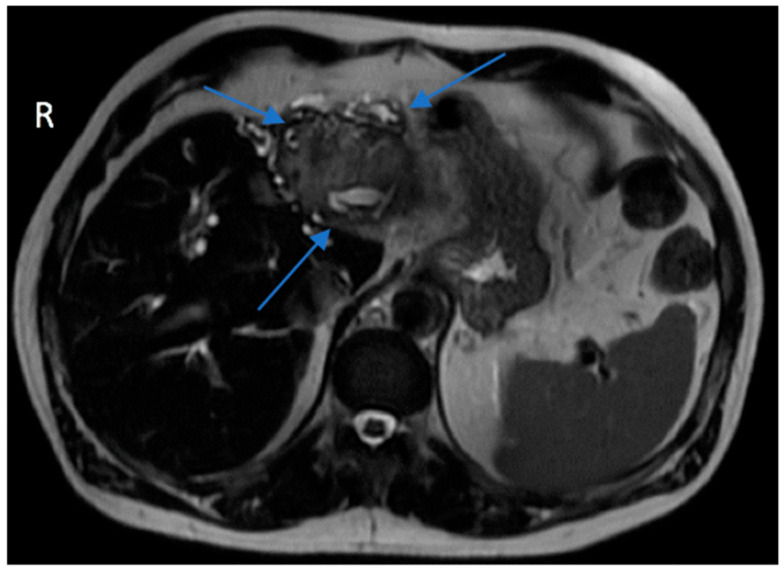
Axial view of the T1-weighted MRI abdomen MRCP with and without contrast showed an intrahepatic cholangiocarcinoma (blue arrows) consistent with the findings on the CT scan. The hypointense lesion extended from the hepatic hilus into the left lobe.

In preparation for the procedure, it was noted that the patient had endorsed lethargy over the past few weeks. Additionally, preprocedural routine laboratory results were notably abnormal. The patient’s direct bilirubin was measured at 2.93 mg/dL, a marked increase from normal values that range between 0.0 and 0.3 mg/dL. Alanine aminotransferase, aspartate aminotransferase, and alkaline phosphatase levels were also elevated at 213 U/L, 118 U/L, and 239 U/L, respectively. Due to the polypoid and irregular nature of the lesion, his concomitant lethargy, and abnormal laboratory results, a CT scan with contrast of the patient’s abdomen and pelvis was performed, which revealed an intrahepatic cholangiocarcinoma with involvement of the left hepatic lobe (Figure 1) and umbilical induration corresponding to the skin lesion (Figure 2). A magnetic resonance cholangiopancreatography (MRCP) was performed for further characterization of the cholangiocarcinoma. This revealed an intrahepatic cholangiocarcinoma extending from the hepatic hilus into the left lobe of the liver (Figure 3), resulting in both intrahepatic and extrahepatic biliary dilation and thus further supporting the previous CT findings.

**Figure 4:**
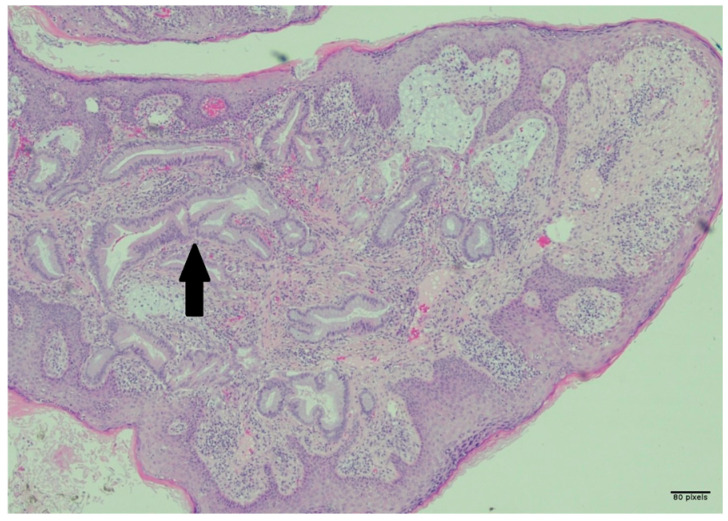
Hematoxylin and eosin (H&E) staining of the patient’s metastatic umbilical lesion. Moderate-power view (×4) showing infiltration of the malignant glands through the papillary and reticular dermis (black arrow). Scale bar = 80 pixels. Findings are consistent with metastatic carcinoma of intrahepatic origin.

An excisional biopsy of the umbilical mass was performed, and the specimen was fixed in formalin. Pathology of the umbilical mass was compatible with metastatic carcinoma. The specimen showed malignant glands infiltrating through the papillary and reticular dermis with nuclear palisading in the metastatic glands, luminal macrophages, associated inflammatory response, desmoplasia, pseudoepitheliomatous hyperplasia of the overlying squamous epithelium, and occasional inflammatory cells infiltrating the epidermis, as seen in Figure 4. Additionally, immunohistochemistry of the mass revealed positive staining for both CK7 and CK19. Further biopsy of the primary tumor was not completed.

Following the procedure and diagnosis of metastatic cholangiocarcinoma, the patient was referred for palliative chemotherapy and biliary stenting at another facility. Further follow-up was unavailable to document the patient’s long-term response.

## 3. Discussion

In this case report, we describe a 65-year-old previously healthy male with approximately one month of fatigue and cholestatic symptoms who presented for an elective umbilical hernia repair. The patient’s abnormal laboratory results, history of fatigue, and atypical umbilical mass increased clinical suspicion for potential malignancy. Further diagnostic imaging and pathology confirmed intrahepatic cholangiocarcinoma involving the left liver lobe with metastasis to the umbilicus, which was initially misdiagnosed as an umbilical hernia. The patient decided to continue his care with another institution in order to manage his symptoms and initiate palliative care.

An SMJN is a rare extra-intestinal manifestation of underlying intra-abdominal and pelvic malignancies that present as umbilical masses [2]. Its detection often indicates an ominous sign of advanced metastasis and is associated with significantly reduced patient survival. A major barrier to early detection lies with both patients and clinicians. The typically benign appearance of the lesion and its infrequent occurrence reduce the likelihood of patients reporting it or clinicians correctly identifying it [10]. Additionally, the lesion may be misdiagnosed as a benign epithelial cyst, umbilical hernia, granuloma, umbolith, or endometrial deposit [10]. Possible primary umbilical malignancies also include malignant melanoma, basal cell carcinoma, and mesenchymal tumors [11]. Because most differential diagnoses for umbilical masses are benign, a false sense of reassurance can occur. Rising rates of intra-abdominal and pelvic malignancies, and their potential extra-intestinal manifestations, should prompt a high index of suspicion for malignancy.

Another major barrier to diagnosing an SMJN is the wide variability in its clinical appearance. Clinically, the lesions are often firm, irregularly shaped masses fixed to the skin [10]. They may range from indurated, painful plaques that are ulcerated or fissured to erythematous, painless, pruritic nodules with a vascular appearance [10,11]. The nodule may occasionally secrete pus, blood, or serous fluid, while others may be devoid of any discharge [12]. In this case study, the patient had a mildly erythematous, polypoid mass with a cauliflower-like appearance. This further emphasizes how the broad variation among lesions complicates the recognition of SMJNs.

Most SMJNs are adenocarcinomas originating from intra-abdominal or pelvic malignancies. While adenocarcinoma is the most common, other primary tumor sources include squamous cell carcinoma, lymphoma, neuroendocrine tumors, and gastrointestinal stromal tumors. Their association with skin metastasis, as seen with SMJN, has been found in only a few cases in the literature [12]. Furthermore, gastric carcinomas are the most common primary tumor sources of adenocarcinoma that are associated with SMJN, accounting for up to 25% of cases, followed by ovarian, colorectal, and pancreatic malignancies [11]. SMJN arising from cholangiocarcinoma remains extremely rare, with only ten prior cases being reported, thus making this case the eleventh [2]. CCA metastasis often occurs via lymphatic or hematogenous routes, and the abundant vascular and lymphatic supply of the umbilicus may facilitate the spread from the primary tumor [2,9]. This highlights the importance of early recognition of these rare umbilical metastases, which can aid in earlier diagnosis and management of advanced abdominal malignancies.

The prognosis of cholangiocarcinoma remains poor, with an average 5-year survival rate of about 5%, as it is often incurable and largely refractory to both surgical and medical interventions [4]. Although the overall pathogenesis is consistent, differences between intrahepatic and extrahepatic classifications, along with associated infectious and noninfectious etiologies, result in a broad spectrum of clinical behaviors and management pathways. Additionally, the rarity of CCA has limited the depth of available studies and clinical guidance. The rising incidence and mortality associated with CCA underscore the need for increased awareness and further research regarding its diagnosis and treatment.

This case of a Sister Mary Joseph nodule found in the setting of cholangiocarcinoma illustrates the complex interactions between neoplasms and different organ systems. Early recognition of these manifestations can increase clinical awareness and aid in early detection with the goal of improving patient outcomes.

## 4. Conclusions

Sister Mary Joseph nodules are rare extra-intestinal manifestations indicative of intra-abdominal and pelvic malignancies, including cholangiocarcinoma. This finding suggests advanced disease processes and, oftentimes, a poor prognosis. This case, to the best of our knowledge, represents the 11th reported case of SMJN in the setting of CCA. Furthermore, it emphasizes the importance of early recognition of this skin manifestation that can present with varying characteristics and the need for further research into the complex disease process to improve long-term management and prognosis.

## Data Availability

The data presented in this study are available on reasonable request from the corresponding author. The data are not publicly available due to privacy concerns.

## Abbreviations

The following abbreviations are used in this manuscript:

SMJN	Sister Mary Joseph Nodule
CCA	Cholangiocarcinoma
MRCP	Magnetic Resonance Cholangiopancreatography

## References

[1] Chang J.L., Huang C.J., Tsai Y.C., Chiang N.J., Huang Y.S., Hung S.C., Shan Y.S., Lee G.B. (2024). An integrated microfluidic system for automatic detection of cholangiocarcinoma cells from bile. Lab Chip.

[2] Ismail I.B., Mlika M., Manai G., Rebii S., Karma K., Zoghlami A. (2025). Sister Mary Joseph’s nodule: A rare metastasis of hilar cholangiocarcinoma: A new case report and review of the literature. Helion.

[3] Qurashi M., Vithayathil M., Khan S. (2025). Epidemiology of cholangiocarcinoma. Eur. J. Surg. Oncol..

[4] Braconi C., Patel T. (2010). Cholangiocarcinoma: New Insights into Disease Pathogenesis and Biology. Infect. Dis. Clin. N. Am..

[5] Vithayathil M., Khan S. (2022). Current Epidemiology of cholangiocarcinoma in Western countries. J. Hepatol..

[6] O’Connor-Byrne N., Glavey S., de Freitas D., Quinn J. (2020). Sister Mary Joseph nodule in mantle cell lymphoma. Lancet Oncol..

[7] An Q., Zhou J., Zhu C., Tian J. (2025). Sister Mary Joseph’s Nodule from Fallopian tube cancer. Asian J. Surg..

[8] Palaniappan M., Jose W.M., Mehta A., Kumar K., Pavithran K. (2010). Umbilical metastasis: A case series of four Sister Joseph nodules from four different visceral malignancies. Curr. Oncol..

[9] Wroński M., Kluciński A., Krasnodębski I.W. (2014). Sister Mary Joseph Nodule: A Tip of an Iceberg. J. Ultrasound Med..

[10] Ray R., Baruah T.D., Ravina M., Kumar D., Minz T. (2023). Umbilical nodule—A not always innocuous abdominal finding. J. Cancer Res. Ther..

[11] Sina B., Deng A. (2006). Umbilical metastasis from prostate carcinoma (Sister Mary Joseph’s nodule): A case report and review of literature. J. Cutan. Pathol..

[12] Soares L., Almeida J. (2013). Sister Mary Joseph Nodule and peritoneal carcinomatosis from squamous cell cervical carcinoma. Proc. Obstet. Gynecol..

